# Development of a low-cost congenital abdominal wall defect simulator (wall-go) for undergraduate medical education: a validation study

**DOI:** 10.1186/s12909-023-04929-3

**Published:** 2023-12-15

**Authors:** Gabriel Araújo Medeiros, Igor José Nogueira Gualberto, Carlos Henrique Nascimento Domingues da Silva, Ana Maria Bicudo Diniz, Jan Beatriz Felinto de Santana, Fábio Perecin Volpe, Rahul Gadde, Alessandra Mazzo, Rodrigo Cardoso de Oliveira, Lourenço Sbragia

**Affiliations:** 1 Bauru Medical School, Department of Pediatric Dentistry, Orthodontics, and Public Health, Bauru School of Dentistry, University of São Paulo, Bauru, Sao Paulo, Brazil; 2 Recife Medical School, Federal University of Pernambuco, Recife, Pernambuco Brazil; 3 Division of Pediatric Surgery, Department of Surgery and Anatomy, Ribeirão Preto Medical School, University of São Paulo, Av Bandeirantes 3900, 10th floor, Ribeirão Preto, São Paulo, SP Brazil; 4 Nursing Course, Unifacisa University Center, Campina Grande, Paraíba, Brazil; 5 Division of Pediatric Surgery, Nationwide Children’s Hospital, Columbus, OH USA; 6 Department of Biological Sciences, Bauru School of Dentistry, University of São Paulo, Bauru, Sao Paulo, Brazil

**Keywords:** Simulation Training, Validation study, Low-cost technology, Gastrointestinal system, Cost, Undergraduate

## Abstract

**Background:**

Congenital Anomalies were responsible for 303,000 deaths in the neonatal period, according to the WHO, they are among the world’s top 20 causes of morbidity and mortality. Expensive simulators demonstrate several diseases, but few are related to congenital anomalies. This study aims to develop, validate, and evaluate low-cost simulator models (WALL-GO) of the most common abdominal wall defects, gastroschisis, and omphalocele, to enable diagnosis through an accessible tool with study value and amenable to replication.

**Methods:**

Market research was conducted to find materials to build low-cost models. The researchers built the model and underwent validation assessment of the selected experts who scored five or more in the adapted Fehring criteria. The experts were assessed through a 5-point Likert scale to 7 statements (S1-7). Statements were assigned values according to relevance in face and transfer validities. Concomitantly, the model was also evaluated by students from 1st to 5th year with the same instruments. Content Validity Indexes (CVIs) were considered validated between groups with concordance greater than 90%. Text feedback was also collected. Each statement was subjected to Fisher’s Exact Test.

**Results:**

Gastroschisis and omphalocele model costs were US $15 and US $27, respectively. In total, there were 105 simulator evaluators. 15 experts were selected. Of the 90 students, there were 16 (1st year), 22 (2nd), 16 (3rd), 22 (4th), and 14 (5th). Students and experts obtained CVI = 96.4% and 94.6%, respectively. The CVIs of each statement were not significantly different between groups (*p* < 0,05).

**Conclusions:**

The WALL-GO models are suitable for use and replicable at a manufacturable low cost. Mannequins with abdominal wall defects are helpful in learning to diagnose and can be applied in teaching and training health professionals in developing and low-income countries.

## Introduction

Simulation is a teaching-learning technique with the added possibility of repeating procedures in a controlled environment, free of the patient’s ethical aspects. As such, replicating everyday life situations using simulation applied through a montage of fictitious cases requiring appropriation of different tools, knowledge, and skills defines simulation-based training (SBT).

This appropriation of different tools shows excellent promise for SBT application in healthcare. It is expected that in simple or complex cases, such as venipuncture or orotracheal intubation, repeating the same procedure in actual patients would become unfeasible, due to possible harm. Åsmund S. Lærdal and Peter J. Safar (1970) developed the first mannequin used in clinical practice for SBT, at first for mouth-to-mouth ventilation training, and later improved for chest compression maneuvers [1]. These and other relevant events were essential contributors that fostered technological advances and spurred healthcare communities’ interest and commitment to training their healthcare professionals to ensure patient safety. Moreover, technological advancements have led to the creation of better simulator models with varied designs that can produce odors and secretions and can even undergo complex surgical procedures countless times. Additionally, models capable of relaying real-time changes in blood pressure, heart rate, and other hemodynamic features during simulated procedures like surgeries and drug application are being developed [2–4].

Great thinkers in medical education, seemingly having these possibilities in mind, played the role of rethinking the foundations of traditional curricula governing healthcare training, establishing new principles that would later significantly impact the curricula of several medical schools worldwide [5]. A recent SBT study found encouraging results, with increased self-confidence and enhanced clinical competence [6]. Based on these expectations and on the growing evidence of the effectiveness of SBT, it would play a vital role in a competency-based new curriculum, and objective structured clinical examinations (OSCEs) would serve as a method for evaluating medical performance in clinical practice [5].

Thus, it was necessary to classify the tools into different technological levels to manage investments and simulation tool usage better. For this, the term “fidelity” is associated with the technology applied in the simulator; that is, mannequins that perform cardiorespiratory functions can be considered high fidelity and promote greater veracity to SBT [7]. However, low-fidelity mannequins help train simple skills, such as intramuscular drug application routes or clinical reasoning exercises [8, 9]. Another essential term is “complexity”, which represents the requirement of prior clinical knowledge in SBT [9].

In addition to the possible methods and applications of SBT, we also define the most common abdominal wall defects (AWD), which are gastroschisis (GS) and omphalocele (OC). In general, the incidence of GS is approximately 1 per 2000 live births, and OC is almost 1 per 4000 [10]. GS is a congenital anomaly represented by incomplete closure of the abdominal wall (usually to the right side of the umbilicus) and, consequently, exposure of the fetal intestine to the uterine cavity and, therefore, to the amniotic fluid [11]. OC consists of an umbilical cord defect in which the intestinal contents do not return to the abdominal cavity after physiological herniation. In most cases, both conditions are visually distinguished by inspection, but a comprehensive fetal ultrasound is required after prenatal OC diagnosis for further investigation of associated syndromes. In up to 49% of diagnoses, this anomaly manifests with chromosomal abnormalities, primarily trisomies of 13, 18, and 21 [12, 13].

As simulators are often expensive and inaccessible in most low- and middle-income countries, this study proposes to develop, validate, and evaluate the low-cost WALL-GO models for recognition and diagnostic training of most common AWD, specifically cases of GS and OC to facilitate the use of manufacturable simulators as teaching and training tools for medical students and professionals alike and for better reception of newborns affected by neonatal diseases.

## Materials and methods

### Design

**Fig. 1:**
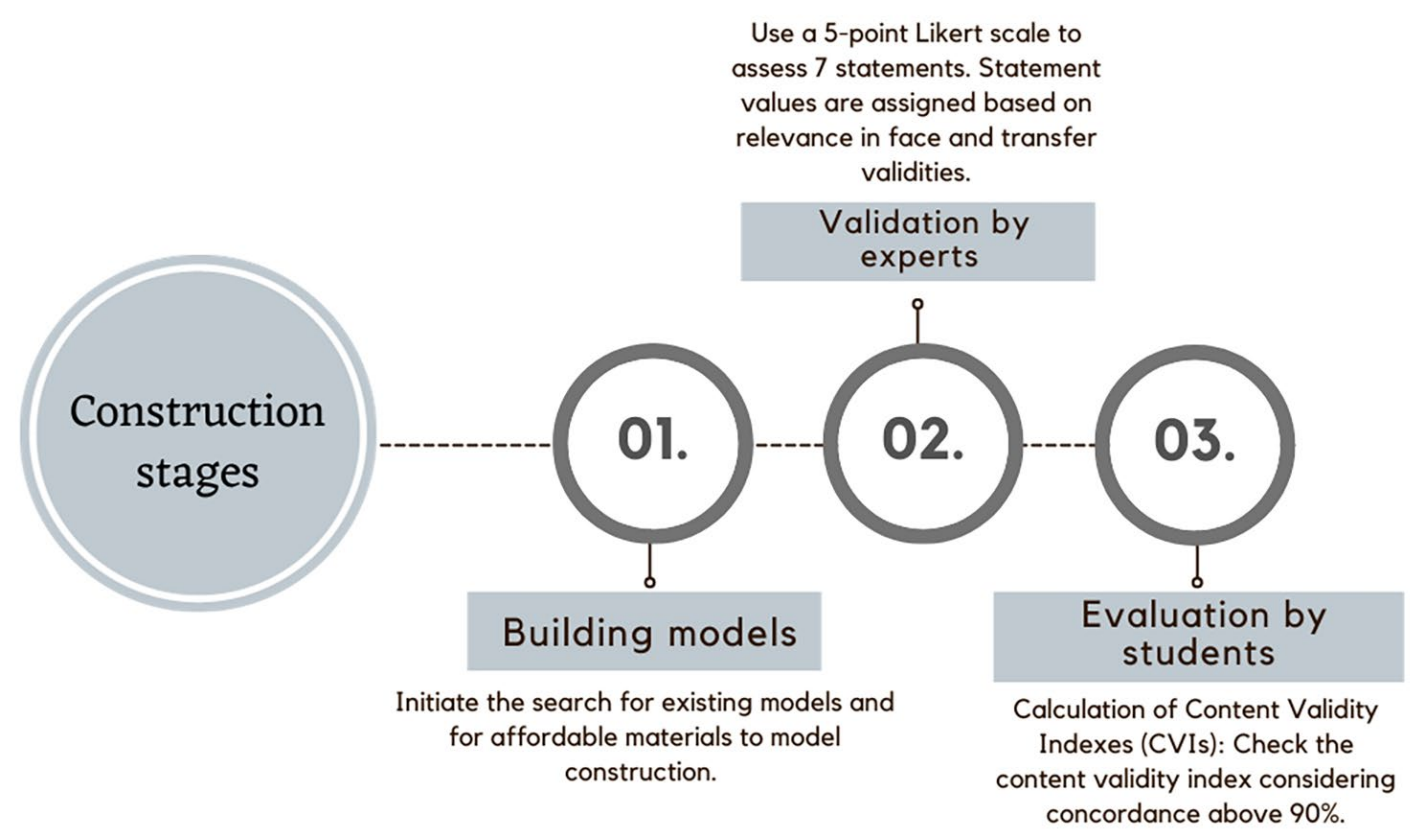
Flowchart representation of the three-stage study Source: self-authored

This is a methodological study of constructing and validating a low-cost simulator for diagnostic training of GS and OC. This methodological research involves three processes: 1 - development, production, and construction of technologies; 2 - validation of technologies; and 3 - evaluation or application of technologies, as proposed by Polit and Beck [14]. This study will, therefore, be presented in three phases, as shown in Fig. 1 (Fig. 1).

As this study presents some qualitative aspects, we aimed to follow the Consolidated Criteria for Reporting Qualitative Research (COREQ) checklist [15]. Only items 24 and 25 could not be covered because the researchers coded the data, and a coding tree was unnecessary.

### Model development

#### First phase: planning, research, and construction of the simulator

For the project development, during a team meeting, the researchers stipulated that the prerequisites for the construction of the simulator would encompass the capability of the constructed simulator to (a) represent a newborn; (b) demonstrate the presence of the umbilicus; (c) demonstrate the AWD; (d) demonstrate the exposed abdominal loop; (e) demonstrate the differences between GS and OC. For this, we prioritized materials that were available in the local market.

An extensive search was conducted by GAM and IJNG on Google between September 7th and September 10th of 2022 to analyze the costs and materials used in the simulators already available on the market. Keywords used were “Gastroschisis”, “Omphalocele”, “Simulator”, “price” and “cost”. The lowest costs were found to be US$324.95 for a moulage to simulate GS and US$ 513.95 for a complete OC simulator model. In order to build the simulator, the first step was the acquisition of two dolls representing children in the neonatal period. In each of them, a circular incision was made, located on the right side of the abdomen of the GS representative, at the level of the navel, with a diameter of 4 cm, and in the other, in the umbilical region, with a diameter of 4 cm, which was the OC representative. These procedures were conducted by LS (PhD, pediatric surgeon, male).

Once this was done, the next step was to find a material whose shape, consistency, malleability, and color were similar to the intestinal loops for their representation on both mannequins. Next, a material covering these loops was needed to represent the membranous sac found in OC. Finally, we sought valuable components for elaborating the umbilical cord, separated from the bowels in GS.

After the construction of the AWD, it was necessary to look for cosmetics that, when applied to the mannequins, would make them resemble children who had just been born.

#### Second phase: validation of the simulator

After its construction, the prototype was validated by a group of experts, invited by convenience, and selected by the researchers after filling out a socio-academic-professional instrument, according to criteria adapted from Fehring [16]. The experts were invited by email, personally or by telephone.

#### Selection and description of experts according to fehring classification

Experts were selected according to whose profile was compatible with a minimum score of five points (Table 1). The physicians who did not reach the minimum score of five points were excluded, as proposed by Fehring. The curriculum vitae (CV) registered on the Brazilian Lattes platform (where you can find free and public information about the CV) was requested to fulfill the criteria (Table 1).

**Table 1:** Criteria proposed by Fehring adapted for expert selection

ADAPTED CRITERIA	POINTS
Master’s Degree *	2
Doctorate Degree *	4
Degree thesis in Master’s or in Doctorate (PhD) **	1
Publication of papers in reference journals (JCR impact factor > 3) **	4
Experience in the teaching field**	2
Clinical experience **	1

*Only the areas of neonatology, pediatric surgery, and maternal-fetal medicine were considered. **Only those related to the topics of simulation as GS and OC were considered

Sixteen physicians were invited to participate in the expert selection process to validate the proposed simulator. Of these, 14 (87.5%) were PhDs, one (6.25%) had a master’s degree, and one (6.25%) had no graduate degree. Considering the topics “simulation”, “gastroschisis”, or “omphalocele”, four items were evaluated: the theme of the graduate dissertation; publication of a paper in a reference journal; teaching experience; and clinical/surgical experience. Only two (12.5%) had a graduate dissertation on one of the three topics. Regarding publishing papers in reference journals, six (37.5%) had published at least one paper, while 10 (62.5%) had not. In addition, 14 (87.5%) had teaching experience, while two (12.5%) did not. Finally, 15 (93.75%) claimed clinical or surgical experience, while one (6.25%) did not.

**Fig. 2:**
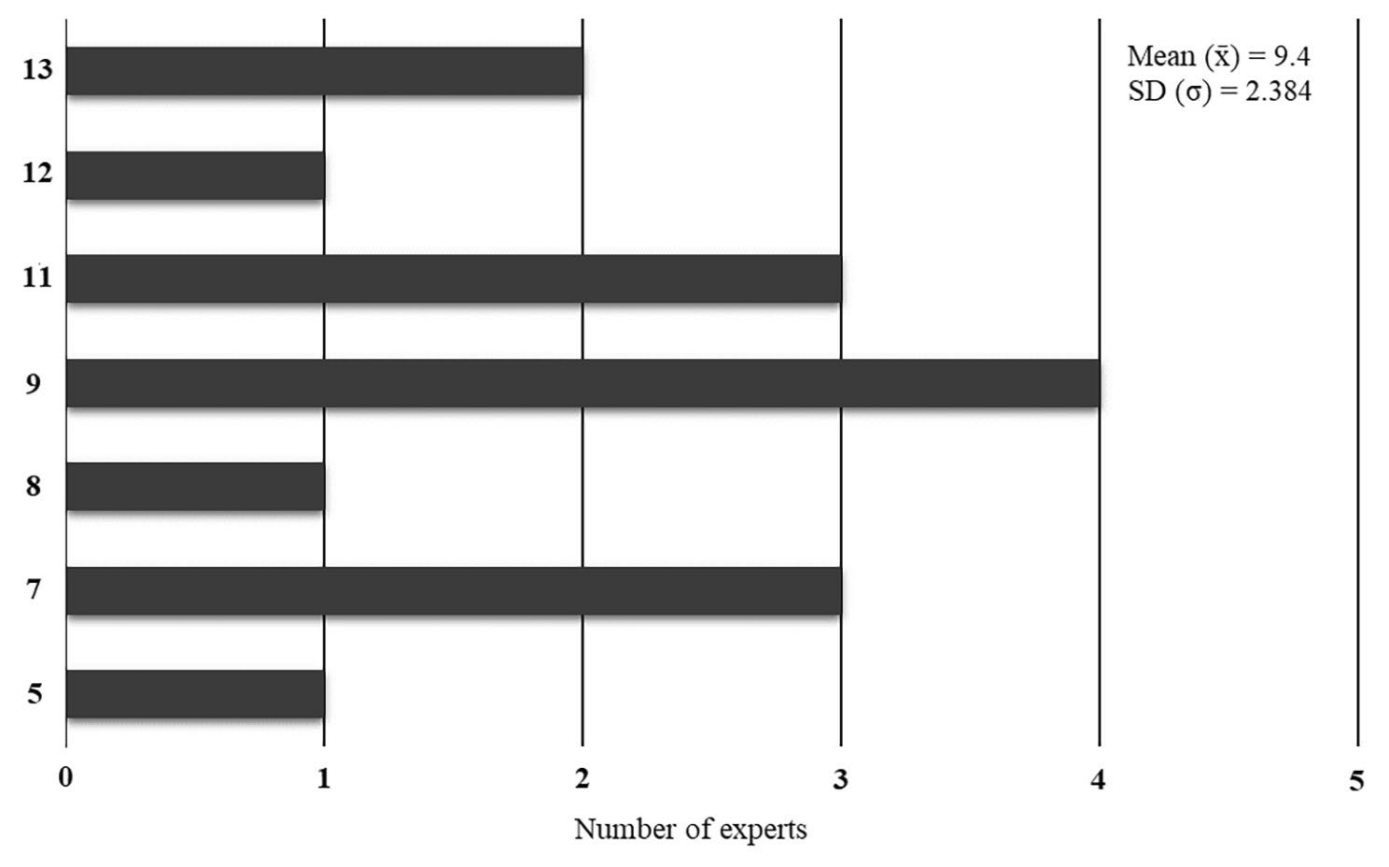
Scores of the selected experts

With these data, 15 (93.75%) physicians (four females and 11 males) numbered 1 to 15 were selected as experts for validating the simulator since they scored at least five points in the Fehring Classification. The specific scores of the selected experts are somewhat high (Mean = 9.4) and homogeneous (SD = 2.384), as described below in Fig. 2. This means that this group is expected to be judicious in the validation process and justifies the division into two subgroups (Experts A and Experts B) to assess whether there is also homogeneity between those who scored more and less according to the adapted Fehring criteria. Experts A contained those above the mean score, and Experts B contained those below the mean score (Fig. 2).

#### Instrument development for validation and evaluation

Furthermore, for the validation and evaluation of the simulator, a psychometric scale developed by the researchers themselves was used, based on the Likert Scale [17], applied to seven statements (S1-7) elaborated to encompass relevant aspects to attest to the quality of the simulator, weighted according to their relevance. The original instrument contained only the statements and prenatal umbilical and paraumbilical ultrasound scan images of GS and OC cases. Those were displayed in “.gif” format for visual comparison with the models and accessed through hyperlinks [18, 19]. As the experts were not in person to perform the validation, an adapted version containing photographic images of the mannequins was also developed.

The instrument for evaluating the simulator was set in a 5 × 7 table. The column had seven questions with assigned value (AV): S1) Prenatal ultrasound information favors the recognition of abdominal wall defects (AV = 1); S2) It is possible to recognize the intestinal loops in the abdominal wall defects on mannequins A and B (AV = 2); S3) The representation of the mannequins favors the recognition of the defects (AV = 2); S4) The abdominal defect in the case of GS is well presented (AV = 2); S5) The umbilical defect in the case of OC Is well presented (AV = 2); S6) The simulator allows training in the diagnosis of GS (AV = 3) and S7) The simulator allows training in the diagnosis of OC (AV = 3). The rows had five answers: (A) Strongly Agree, (B) Agree, (C) Undecided, (D) Disagree, and (E) Strongly Disagree.

The results collected through the questionnaire were analyzed from the premises of disagreement and agreement. The marks with “Strongly Disagree”, “Disagree”, and “Undecided” were considered disagreement, and the marks with “Strongly Agree” and “Agree” were considered agreement. Based on these two premises, the Content Validity Index (CVI) was calculated as a criterion for comparing each item among respondents. [20]. The formula to calculate CVI is as follows: CVI = Total number of concordant/total number of answers. A CVI ≥ 0.9 was considered satisfactory, i.e., when 90% or more of the participants agreed with the item. It is worth mentioning that to calculate the overall CVIs of the students and the experts, the assigned value average of the CVIs of each item was used.

#### Third phase: evaluation of the simulator

After the validation with experts, the prototype was submitted to an in-person evaluation of the simulator, with the original instrument described in the Second phase, this time applied by GAM (an inexperienced male medical student) with the supervision of LS, to 90 students enrolled between the first and fifth years of medical schools from two campuses of the University of Sao Paulo, one located in Bauru city (60 students) and the other in Ribeirão Preto city (30 students), who were numbered from 1 to 90 according to the time sequence of the responses. Before this submission, the students had a brief theoretical exposition in their university’s domain that covered the essential aspects of identifying GS and OC in newborns, delivered by LS. They were invited to participate through email, and all data were collected in the same room where the exposition occurred. Three 1-hour meetings were held; the first took place in Bauru and had 40 participants; the second took place in Bauru with 20 participants; and the third took place in Ribeirão Preto with 30 participants. The students’ evaluations were also analyzed by their CVI. Regarding the relationship between the participants and GAM, a few shared little acquaintance with each other, but most students had no relationship with the interviewer. No further information, such as reasons and interests in the research topic, was provided about the researchers.

### Statistical analysis

All data were analyzed to find associations between different expertise groups (Experts A, Experts B and Students) and CVI using the Fisher’s Exact Test. Expert score descriptive data display was defined after the Kolmogorov-Smirnov and Shapiro-Wilk tests evaluated normality. The Statistical Package for the Social Sciences (SPSS version 25.0, SPSS, Inc., Chicago, Illinois, USA) was used in all analyses. A *p*-value of 0.05 was considered statistically significant.

## Results

The materials used to build prototypes that simulate GS and OC were a 40 cm doll, latex tourniquet tube, sausage (≈ 25 cm), pink fabric tape, female condom, yellow cellophane paper, umbilical cord clamps, fake blood makeup, talcum powder. The total material cost was US$ 42.00 (US$ 21.00/manikin), all available in the local market (Table 2).

**Table 2:** Materials, measurements, and price of the simulators

MATERIAL	MEASUREMENT	PRICE
Vinyl doll (n = 2)	40 cm length	20.00 US$
Latex tourniquet tube	5 mm x 1 m	4.00 US$
Sausage	15 mm x 15 cm	0.50 US$
Pink fabric tape	48 mm x 1 m	6.00 US$
Female condom (n = 3)		3.00 US$
Yellow cellophane paper	0,5 m²	0.50 US$
Umbilical cord clamp		1.00 US$
Fake blood makeup	20 ml	4.00 US$
Talcum powder	100 g	3.00 US$

The total cost of the materials was $42.00, of which US$15.00 was for the GS and US$27.00 was for the OC model. Conventional items were also used, such as scissors, glue, blue and red pens, and white correction fluid

**Fig. 3:**
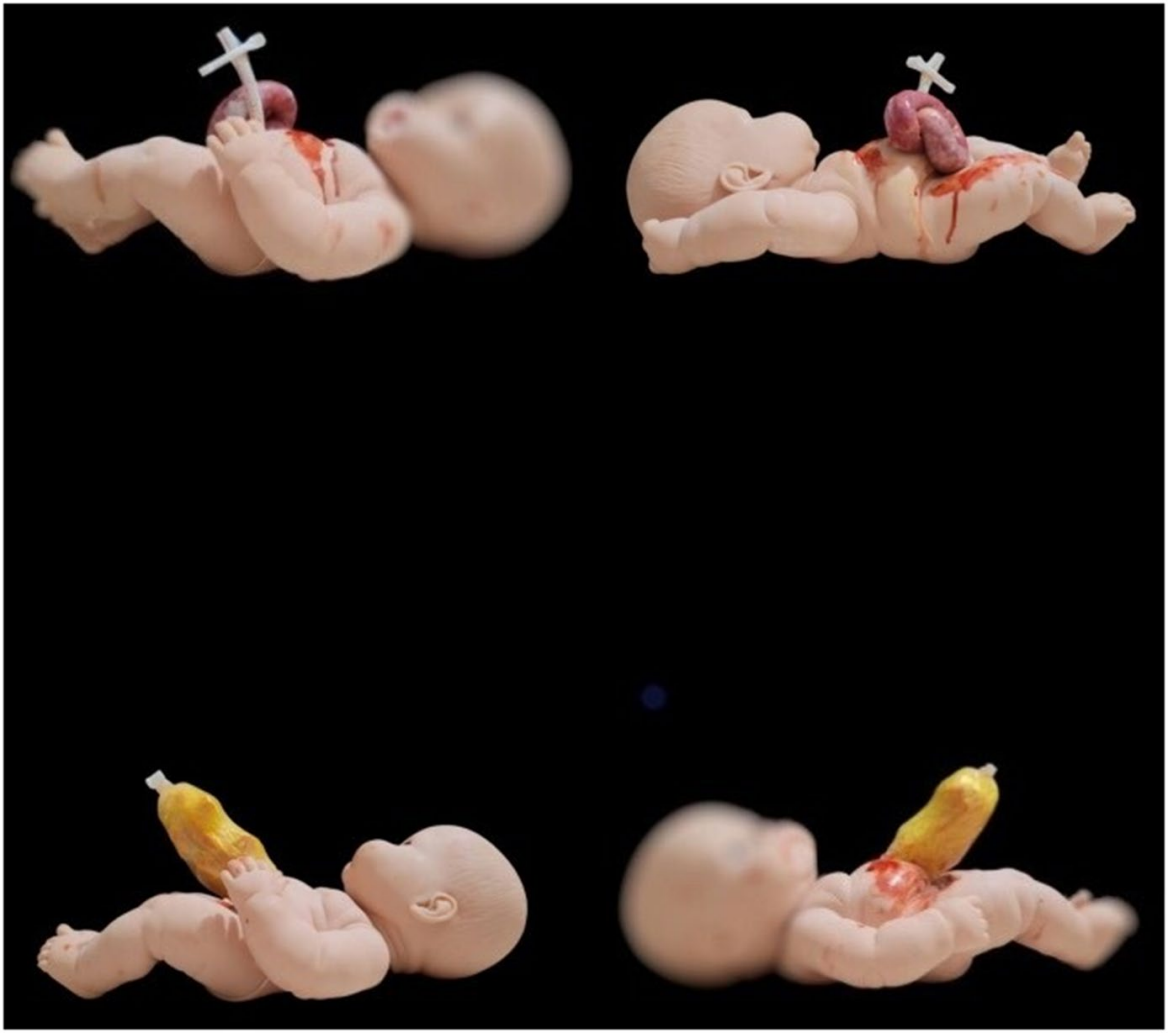
Photographs of the finished GS (up) and OC (down) mannequins in a lateral plane

**Fig. 4:**
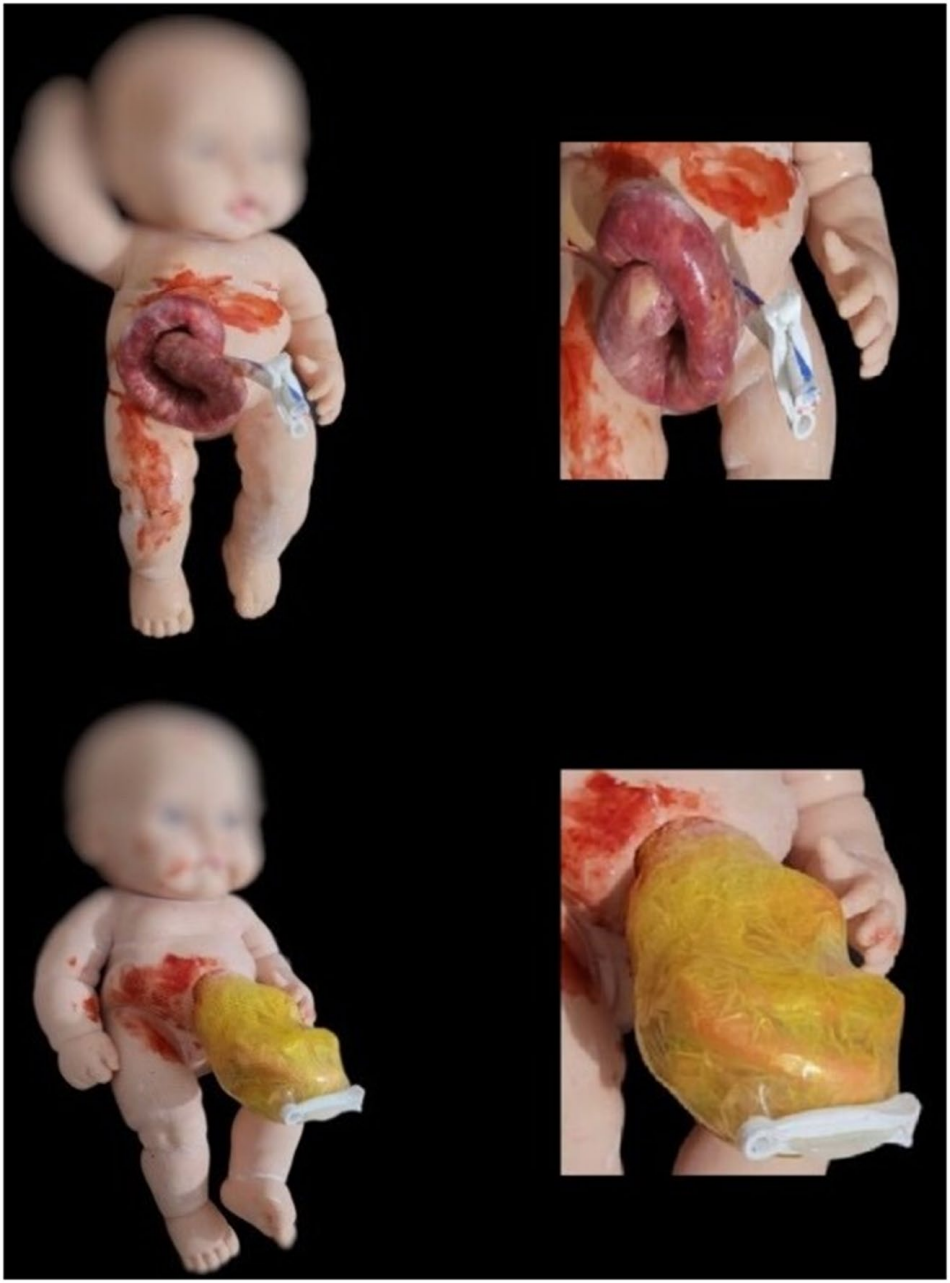
Photographs of the finished GS (up) and OC (down) mannequins in the frontal plane

Display of materials used and final construct (Figs. 3 and 4).

### Validation and evaluation results

**Fig. 5:**
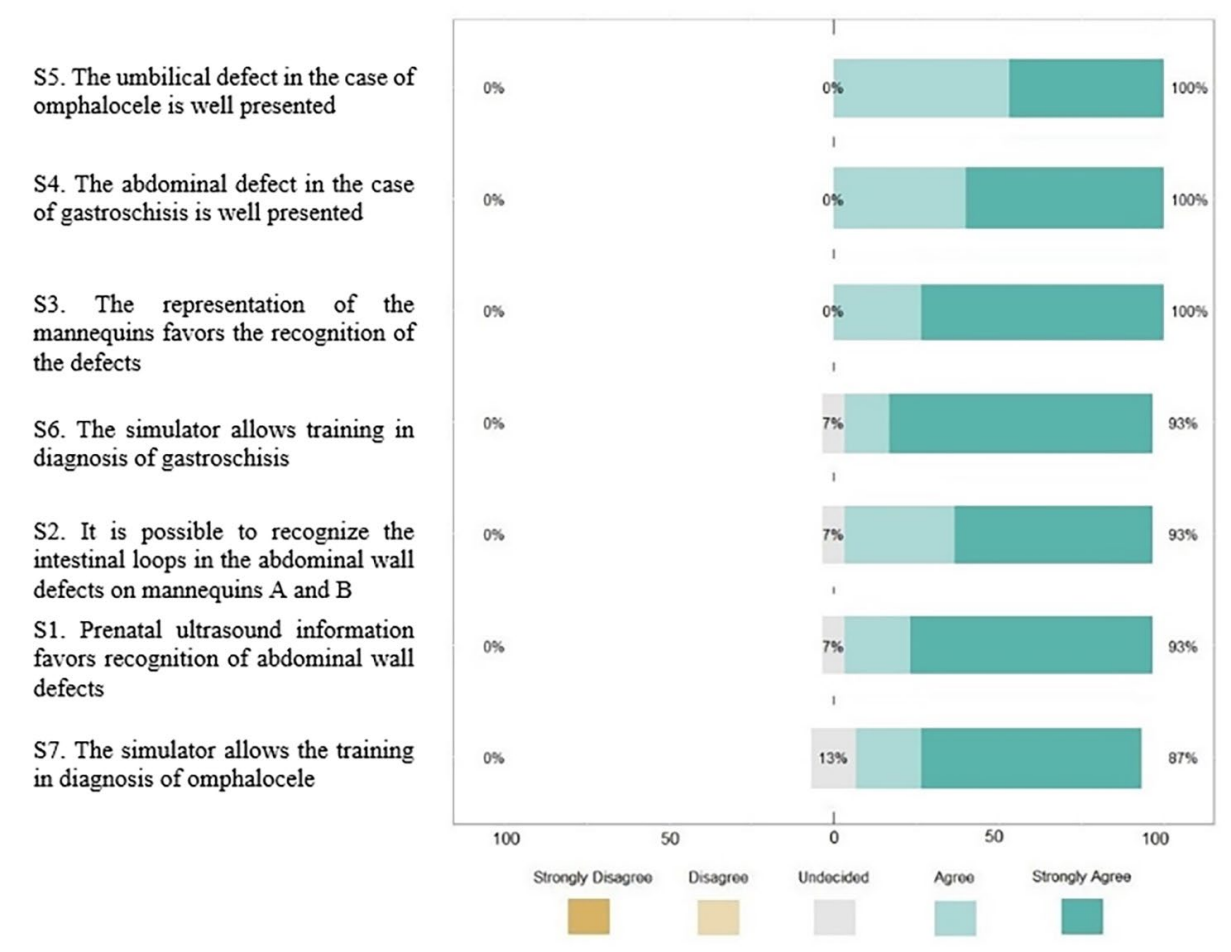
The experts performed the validation results

**Fig. 6:**
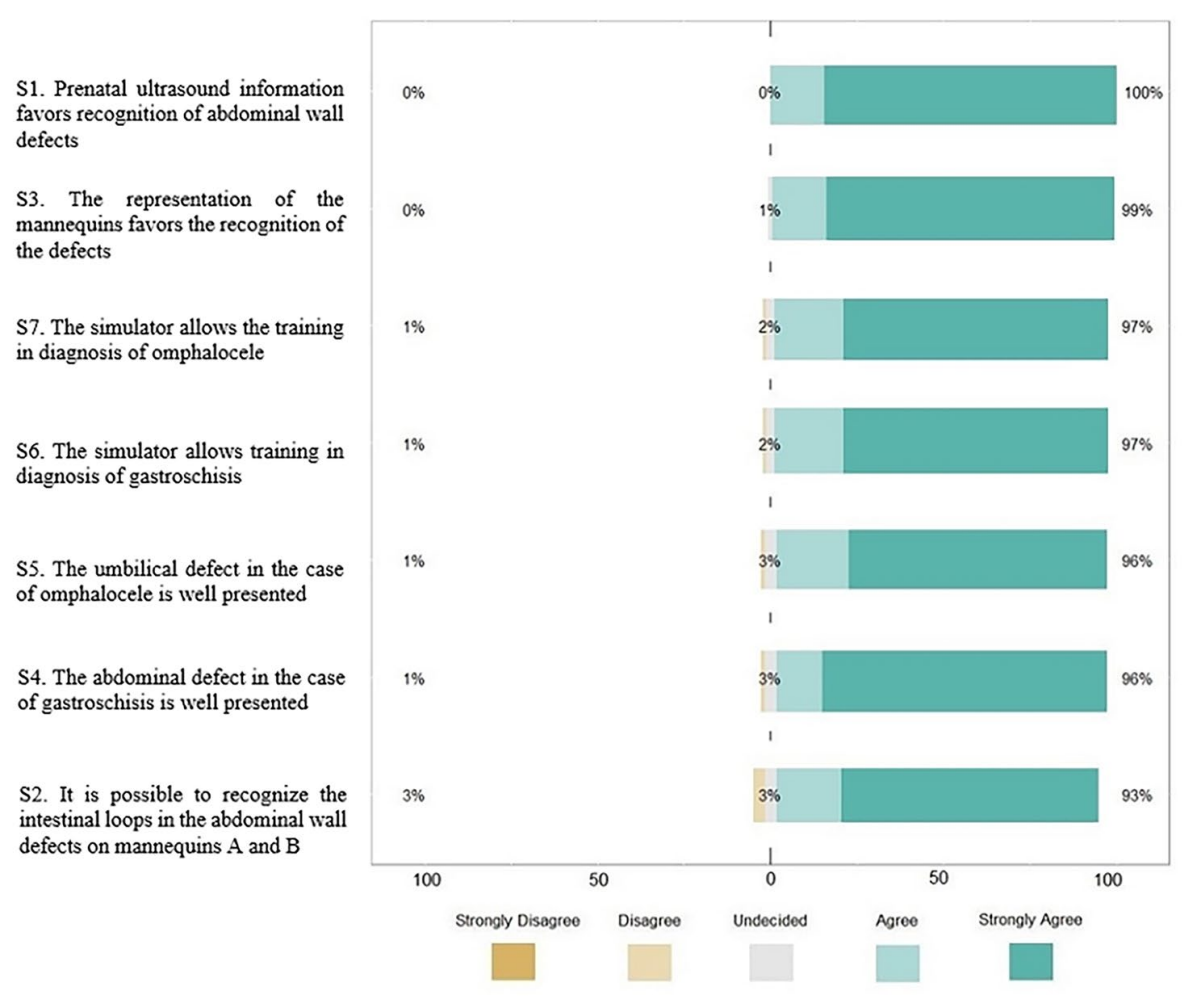
The students performed the evaluation results

The study had 105 participants. There were 15 experts, of which six (two females and four males) were in group A and nine (two females and seven males) were in group B. In the evaluation, 43 were female and 47 were male. The answers for each item in the questionnaire were organized into two graphs plotted on the Likert Scale, in which it is possible to identify all the (dis) agreement premises and their percentages in decreasing agreement order. The first one describes the responses per item of the experts (Fig. 5). The second describes the responses per item of the students from Bauru along with those from Ribeirão Preto (Fig. 6).

Using the data presented in these two graphs, it was possible to calculate the specific CVIs for each item answered and the overall CVIs for the students and the experts when considering all items (Table 3). The simulator achieved a CVI greater than 0.9. It is worth noting that students generally assigned higher ratings than the experts and that no significant difference (*p* < 0.05) was observed between the groups, both on individual items and overall, which ensures agreement between the participants.

**Table 3:** Result of the CVIs of the students and Experts

	S1	S2	S3	S4	S5	S6	S7	OVERALL
CVI of Students	1	0.93	0.99	0.96	0.96	0.97	0.97	0.97
CVI of Experts A	0.83	1	1	1	1	0.83	0.83	0.92
CVI of Experts B	1	0.89	1	1	1	1	0.89	0.96
CVI of Experts	0.93	0.93	1	1	1	0.93	0.87	0.95

S1 *p*-value 0.14; S2 *p*-value 0.59; S3 *p*-value 1; S4 *p*-value 1; S5 *p*-value 1; S6 *p*-value 0.46 and S7 *p*-value 0.14 (Fisher exact test)

## Discussion

We validated an AWD simulator. Based on the simulators’ classification, the use of mannequins as low-fidelity and low-cost simulators and their applicability in SBT for both simple and highly complex situations might be promising, such as in peripheral venous access training or differential diagnosis training of visually similar cases, like closed GS and OC, or GS and OC with membrane rupture [21, 22]. Our study could attain some advance in enabling this kind of scenario, for little adaptations could be made to simulate membrane rupture, such as a little incision in the cellophane paper.

Despite the considerable diffusion and success of simulation technology in pediatric procedures, some obstacles still need to be overcome in the recognition and diagnostic training of GS and OC, especially when accounting for the low access and high cost of producing quality simulators [23, 24]. A recent study involving seven low- and middle-income countries (El Salvador, Mozambique, Trinidad, Tobago, Lesotho, Malawi, and Nepal) corroborates this statement. It offered theoretical and practical training for healthcare providers based on low-cost portable simulators. In the study, candidates received SBT on procedures required for screening, diagnosis, and treatment of cervical cancer before performing them in a clinical context. The study included 506 participants, increased confidence in performing the visual inspection after the application of acetic acid, the colposcopy and cervical biopsy, the ablation, and the loop electrosurgical excision procedure was 69%, 71%, 61%, and 76%, respectively [25]. This shows that using low-cost simulators holds promise for usage in SBT and can help aid disease screening and diagnosis even in resource-restricted countries.

Our study is the first to present a validated and low-cost model of OC for SBT. Low and high-cost GS and high-cost OC models have already been developed; however, validation methods were not performed in some of them [26–28]. Similar validation studies were recently conducted to train different abilities [29, 30].

Our purpose was diagnostic training, and since GS and OC are visually distinctive, shape, consistency, malleability, and color similarities were considered when selecting materials. Although, some concerns were reported via feedback: “Sausage is a perishable material” (Student 3); “The cord clamp seems to be too close to the viscera” (Expert 4); “The presence of blood could be confusing in the possibility of bowel damage” (Expert 7); “OC resembles GS with silo correction (Expert 9)”. One comment of significance suggested developing two GS models differentiating inflamed and non-inflamed viscera.

These considerations play an essential role in future studies, as our research does not end with material selection; it might continue after feedback validation through further studies. These considerations characterize participatory action research, which aims to empower the participants to reflect on the produced changes on a subject [31]. The comment on perishable material highlighted the GS model as not reusable, while the OC model can be reused. This way, simple improvements could be considered in the next version(s) of WALL-GO and may include sausage replacement with red colored cotton ball wrapped in cellophane tape, which is non-perishable, moving the cord clamp away from the viscera, and removing blood makeup. Regarding OC resemblance to GS with silo correction, further research is needed to find alternative low-cost materials that can aid with better differentiation for these conditions.

Among physicians, 15 participants passed the Fehring selection method. Since being a graduate was the main criterion for separation between students and experts, conclusions drawn from other fields’ expert-novice studies – a more experienced expert group would better differentiate their opinion from undergraduate students [32]. The Likert score was expected to be lower in the Expert group compared to the students, especially in S6 and S7, based on our assumption that experts would use more technical criteria and consequently have a higher level of demand on the models’ representation of GS and OC. Although the scores for questions S6 and S7 of the experts were indeed lower, even lower in experts A, no statistical difference was observed to attest to any association, in these or in any of the items, which ensures agreement between the participants. Students agreed on the contribution of ultrasound for defect view in the proposed simulation-based training. Students’ and experts’ overall CVI scores passed the criteria (> 0.9). The CVI is among the most reputable instruments to ensure transfer and face validity [33]. Compared with similar studies for validating low-cost simulators in the medical field, we consider the number of experts used in the present study to be satisfactory. The average number of experts used was comparable to other simulator validation studies (11.75 versus 15 in our study) [29, 30, 34, 35].

### Strengths and limitations

Our studies strengths are evident in its innovative and cost-effective design, as evidenced by our simulator model created using scarce and affordable material. Additionally, our methodology incorporated a comprehensive three-stage approach that encompasses simulator development, expert validation, and student evaluation.

The study has several limitations. Firstly, participant selection, most notable in the experts, which was subject to their availability; limits the generalizability of the study’s findings. Next, discrepancy in presentation format (images vs. in-person) between the two groups could influence participant perception and by extension the study’s internal validity. The last concerns our study design, as transfer validity was not fully addressed. Transfer validity is defined as “how the simulator has the effect it proposes to have” [36]; in this context, diagnostic training on AWD, specifically GS and OC. To answer transfer validity, we propose further studies to measure the reproducibility of WALL-GO. The Kirkpatrick four-stage model can be used as an assessment tool, which has been validated and widely used to evaluate training [37]. However, transfer validity can only be addressed with the incorporation of long-term follow-up into the study design and should be considered during future studies.

### Future direction

This study in its’ strengths and limitations can provide the framework for future studies aiming to assess the WALL-GO simulators applicability and its validation across diverse cultural and healthcare settings, ensuring its effectiveness in different educational environments. Importantly, future, and further studies should focus on (1) long-term educational outcomes: assessing WALL-GO simulators impact on clinical decision making and patient outcomes providing valuable insight into the simulators’ effectiveness; (2) continuous improvement: feedback-based development of iterative simulator versions will contribute towards its continued effectiveness and relevance in medical education.

## Conclusion

In conclusion, our study presents a promising, low-cost alternative to address the challenges of diagnostic training in AWD and numerous other pathologies, particularly in resource-limited settings. Moreover, the study exhibits strengths (innovation and methodology) and addresses the inherent limitations, which require further research to enhance the validity and educational impact garnered from the simulator. As such, the WALL-GO simulator promises to be a potentially valuable tool in the evolving simulation-based medical education landscape.

## Data Availability

Data is available upon request from the first author.

## List of Abbreviations

CA: Congenital Anomalies
AWD: Abdominal wall defects
GS: Gastroschisis
OC: Omphalocele
CVI: Content Validity Index
SBT: Simulation-based training
OSCE: Objective structured clinical examination
